# Co-occurring Mental Disorders in Substance Abuse Treatment: the Current Health Care Situation in Germany

**DOI:** 10.1007/s11469-017-9784-5

**Published:** 2017-07-11

**Authors:** Hanna Dauber, Barbara Braun, Tim Pfeiffer-Gerschel, Ludwig Kraus, Oliver Pogarell

**Affiliations:** 1 IFT Institut für Therapieforschung, Parzivalstr. 25, 80804 Munich, Germany; 2 Department of Psychiatry and Psychotherapy, Ludwig-Maximilians-University, Munich, Nußbaumstr. 7, 80336 Munich, Germany; 3 Centre for Social Research on Alcohol and Drugs (SoRAD), Stockholm University, 10691 Stockholm, Sweden

**Keywords:** Substance use disorder, Mental disorders, Comorbidity, Treatment, Health care

## Abstract

Aim of this study was to investigate the current health care situation for patients with co-occurring mental disorders in addiction treatment. Therefore, data from the German Substance Abuse Treatment System (*N* = 194,406) was analysed with regard to the prevalence of comorbid mental disorders, treatment characteristics and outcomes of patients with comorbid psychiatric diagnosis. In outpatient setting, the prevalence of comorbid diagnoses was considerably lower (4.6%) than in inpatient setting (50.7%), but mood and anxiety disorders were the most prevalent additional diagnoses in both settings. In the treatment of patients with these comorbid disorders, we found higher rates of complementary internal and external (psychiatric) treatment, more co-operations and referrals after treatment, and positive treatment process outcomes. Findings indicate that the knowledge of an additional diagnosis influences the health care provision of affected patients and can therefore be seen as the essential precondition for providing adequate and comprehensive treatment. This highlights the importance of a sufficient consideration and diagnostic assessment of mental disorders in addiction treatment to further improve the health care situation of comorbid patients.

Substance use disorders mostly co-occur with a multitude of other physical and mental diseases. Studies have consistently shown the high co-occurrence and the elevated risk of mental disorders in individuals with substance use disorders (Baldacchino and Corkery 2006; Flensborg-Madsen et al. 2009; Kessler et al. 1996, 2005; Lieb et al. 2010), which can especially be observed in clinical samples. About two thirds of all alcohol or drug-dependent patients in substance abuse treatment show at least one additional current mental disorder, whereas the lifetime prevalence ranges up to 90% (Adamson et al. 2006; Chan et al. 2008). Thereof mood and anxiety disorders are the most prevalent (Adamson et al. 2006; Langas et al. 2011; Lyne et al. 2011; Swendsen and Merikangas 2000; Schneider et al. 2001; Verheul et al. 2000).

The co-existence of a mental disorder can highly interfere with the treatment of the substance use disorder and is associated with a higher psychopathological severity (Ringen et al. 2008), repeated relapse (Mazza et al. 2009; McCarthy et al. 2005), poorer treatment outcomes (Boden and Moos 2009; Compton et al. 2003; Mueser et al. 2003) and generally poorer prognosis for the individual (Brown et al. 2004; Cacciola et al. 2001; Drake and Wallach 2000; Friedmann et al. 2003; Johnson 2000), thus posing the question for adequate treatment. In recent years, an integrated treatment has been clearly proven to be the most effective approach to treat comorbidity (Brousselle et al. 2010; Brunette and Mueser 2006; Drake et al. 2004; Minkoff and Cline 2004; Moggi et al. 2002; RachBeisel et al. 1999; Mueser et al. 2003) and is therefore recommended in practice guidelines, also in Germany (AWMF 2015; Center for Substance Abuse Treatment 2005). But in spite of the evidence and this recommendations, the fact that in Germany and in most other European countries mental health and addiction care operate quite separately (Baldacchino and Corkery 2006; Ness et al. 2014) causes delay in the implementation of this knowledge into practice and constitute a barrier in recovery. Hence, individuals with co-occurring mental disorders are often faced with considerable difficulties when trying to get adequate treatment for their multiple problems.

## Addiction Care

Within the German Addiction Treatment System, the heterogeneity of services and the associated differences in their structures might often impede an adequate consideration of additional mental disorders. Accordingly, differences in personal resources and qualification of staff, legal frameworks and demand of service providers are related to varying assignments and treatment offers, which range, among others, from giving mere information over counselling to providing treatment (DHS 2014). This is consistent to the finding that routine assessment for psychiatric disorders does not always form part of the standard diagnostic procedures performed at the commencement of treatment in drug services (EMCDDA 2013). Accordingly, it can be assumed that especially in outpatient setting, where a diagnostic assessment of additional mental (or other) disorders is generally not intended or required, limited resources like the low percentage of medical and therapeutic staff (Braun et al. 2014e–f) may lead to an underdiagnosing and neglect of comorbid mental disorders. This assumption is supported by the fact that in outpatient setting only 4.6% of all patients have a documented additional psychiatric diagnosis (Braun et al. 2014e). Contrastingly, in inpatient setting and in structured outpatient rehabilitation treatment, where a diagnostic assessment is structurally intended and specialised personnel resources like psychiatrists, psychologists or medical doctors are available to ensure a diagnostic assessment within treatment facilities, the prevalence of comorbid psychiatric diagnoses is much higher (over 50%) (Bachmeier et al. 2015; Braun et al. 2014f; Fachverband Sucht e.V. 2014; T. Wessel, personal communication, December 17, 2015) and comparable to empirical findings. Nevertheless, it is important to investigate those patients with a documented comorbid diagnosis, even in outpatient setting, to determine how a known comorbid diagnosis affects treatment planning, course and outcome of treatment and the provision of complementary health care.

## Aims

Against the background of an international dialogue on effective treatment of co-occurring substance and mental disorders and the known barriers and straits in providing adequate treatment (McGovern et al. 2006), an improved knowledge of the current health care situation for individuals with comorbidity entering addiction care seems worthwhile. Thus, an overview of available structures and treatment approaches can have important implications for health care planning and prevention. To date there have been only a few studies in Germany investigating the health care situation for people with comorbidity. Therefore, we analyse data from the large national monitoring system of addiction treatment centres in Germany, which can provide insight into the management of comorbid disorders in practice. In particular, we want to analyse these data exploratory with regard to (a) the prevalence of comorbid psychiatric diagnoses, (b) course of illness and treatment in patients with comorbid disorders, (c) treatment characteristics and process outcomes of patients with comorbid disorders and (d) relevant structures in the supply of individuals with comorbidity. Furthermore, sociodemographic parameters of patients with a comorbid psychiatric diagnosis will be outlined to mark special characteristics of this clientele. As mood and anxiety disorders constitute the most common comorbid disorders, we will investigate in particular the sample of patients with these comorbid diagnoses. Overall aim of this investigation is to gain insight how the comorbidity problem is actually addressed in German addiction care and thereby derive practical recommendations to optimise the supply situation for people with comorbidity.

## Methods

### Design and Sample

Data from individuals within the German Addiction Care System (*n* = 822 outpatient centres, *n* = 200 inpatient centres), which is documented annually within the national monitoring system (Statistical Report of Substance Abuse Treatment; DSHS; actual: Brand et al. 2015) and provides information on treatment facilities as well as on sociodemographic, disorder and treatment related characteristics of patients, was analysed descriptively. We analysed data of patients with a documented additional ICD-10 F3x (outpatient: *n* = 2673, inpatient: *n* = 6853; Braun et al. 2014a, b) or F4x diagnosis (outpatient: *n* = 1130, inpatient: *n* = 3430; Braun et al. 2014c, d), which have entered addiction treatment in 2013. For comparison, the total sample of *n* = 154,344 cases in outpatient and *n* = 40,062 cases in inpatient setting (further referred to as “total sample”), which are documented within the DSHS, was analysed (Braun et al. 2014e, f). The following main substance diagnosis groups (MD) were included in the analysis: alcohol, opioids, cannabis, sedatives, cocaine, stimulants and other psychotropic substances. For data protection reasons, cases are aggregated on a facility level and no individual data is available. For further specifications of the methods, see the actual report of the DSHS (Brand et al. 2015).

### Instruments

Data is documented with the German Core Data Set (KDS; Deutscher Kerndatensatz zur Dokumentation in der Suchthilfe; DHS, 2010) within the treatment centres. Documentation is carried out via computerised software systems. The diagnostic of the KDS is based on the International Classification of Mental Diseases (ICD-10; Dilling et al. 2013) and is intended to be carried out uniformly across treatment centres at beginning and end of treatment. Besides substance use disorders, also comorbid psychiatric disorders are documented according to ICD-10, either if a psychiatric diagnosis already exists at the beginning of treatment (mainly in outpatient setting) or after a diagnostic assessment within the facility (mainly in inpatient setting). For a comprehensive description of the variables and item categories, see the KDS manual (DHS 2010).

### Measures

Prevalence rates are based on reported (Fx) diagnoses, assessed by the KDS variables “main (substance) diagnosis” and current “further psychiatric, neurologic and other diagnosis”. The sample of patients with additional F3x (further referred to as mood disorders) and F4x diagnoses (further referred to as anxiety disorders) was examined with regard to the sociodemographic parameters gender, age, relationship status and employment status. Course of illness and treatment were measured by the items duration of the substance use disorder and previous treatment. The variables length of treatment, internal treatment (within the facility) and external treatment (in other facilities) were used to describe treatment characteristics. Treatment process outcomes were analysed by the items treatment termination (“regular”/“irregular”, with “regular” comprising the regular termination of treatment, termination due to the instigation or with the consent of the therapist or the regular transition to another service, and “irregular” comprising premature termination due to disciplinary reasons, patients choice, unscheduled transition to another service or the death of the patient) and treatment outcome assessment (“positive” (successful or improved) or “negative” (steady or impaired) assessment of the substance related problems after end of treatment). For analysis of supply structures, the variables co-operation with (other services), referral (into treatment) and referral (after treatment) were used.

### Analysis

A descriptive data analysis was conducted. Due to structural differences (see “Addiction Care”), results are reported separately for outpatient and inpatient treatment setting. For comparison, outcomes are reported for the total sample of patients. Because of the aggregated data format, it is not possible to use common statistical tests to test for mean differences. Furthermore, the use of *χ* ^2^ tests is not recommended in such large sample sizes due to the high sensitivity. Even small differences in the frequency distribution may reach statistical significance, without being clinically significant, misleading the interpretation of results (Bortz 2005). Therefore, a descriptive analysis can be considered as more sensible. Especially in view of the large size of the data set and the widespread coverage of treatment centres and the therefore highly representative results for all patients in German outpatient (estimated attainment rate about 70%) and inpatient centres (estimated attainment rate about 60%) (Brand et al. 2014), an exploratory investigation of these data seems appropriate.

## Results

### Prevalence of Comorbid Psychiatric Diagnoses

The overall prevalence of additional psychiatric diagnosis was 4.6% in outpatient and 50.7% in inpatient setting. Among all patients with a documented additional psychiatric (ICD 10 Fx) diagnosis, mood disorders (outpatient, 45.7%; inpatient, 41.0%), personality disorders (outpatient, 21.0%; inpatient, 25.4%) and anxiety disorders (outpatient, 19.0%; inpatient, 20.3%) accounted for the most frequent comorbid diagnoses (see Table 1). With regard to the main substance diagnosis groups, we found a divergent distribution of comorbid psychiatric diagnosis (see Table 1). In patients with problematic use of alcohol or sedatives, we found the highest percentage of mood (outpatient: 52.2% resp. 39.9%, inpatient: 45.3% resp. 36.4%) and anxiety disorders (outpatient: 20.0% resp. 30.8%, inpatient: 21.2% resp. 39.9%), whereas in patients with cannabis or stimulants use disorders, we found a comparatively high proportion of schizophrenic disorders (outpatient: 14.8% resp. 14.7%, inpatient: 10.6% resp. 8.9%) and disorders with onset in childhood and adolescence (outpatient: 9.4% resp. 11.5%, inpatient: 14.8% resp. 15.2%). Patients with cocaine or opioid dependence showed the highest level of personality disorders (outpatient: 30.1% resp. 38.9%, inpatient: 36.3% resp. 37.0%) and patients with problems due to other psychotropic substances also showed a high amount of schizophrenic disorders (outpatient, 20.3%; inpatient, 12.5%) and disorders with onset in childhood and adolescence (inpatient, 13.9%).

**Table 1:** Proportion of additional (ICD-10 Fx) diagnoses among all patients with documented comorbid diagnosis, MD groups, *percentage*

		F0x	F2x	F3x	F4x	F5x	F6x	F7x	F8x	F9x
Alcohol	Outpatient (*n* = 3921)	1.3	3.7	52.2	20.0	1.3	17.9	1.6	0.6	1.5
	Inpatient (*n* = 14,595)	1.3	2.3	45.3	21.2	2.2	23.4	1.6	0.6	2.1
Opioids	Outpatient (*n* = 545)	4.2	9.7	33.0	15.0	0.7	30.1	0.4	0.7	6.1
	Inpatient (*n* = 793)	0.4	5.2	31.4	18.4	1.8	36.3	0.4	0.5	5.7
Cannabis	Outpatient (*n* = 671)	0.7	14.8	32.5	15.2	1.8	23.1	1.6	0.9	9.4
	Inpatient (*n* = 1272)	0.5	10.6	25.2	15.6	1.7	30.5	0.7	0.5	14.8
Sedatives	Outpatient (*n* = 143)	1.4	5.6	39.9	30.8	1.4	20.3	0.0	0.0	0.7
	Inpatient (*n* = 338)	0.6	0.9	36.4	39.9	3.0	18.3	0.0	0.0	0.9
Cocaine	Outpatient (*n* = 131)	0.0	1.5	37.4	17.6	0.0	38.9	0.8	0.8	3.1
	Inpatient (*n* = 208)	0.5	6.7	31.7	11.1	3.4	37.0	0.5	0.0	9.1
Stimulants	Outpatient (*n* = 278)	1.8	14.7	24.8	14.7	0.7	29.1	1.8	0.7	11.5
	Inpatient (*n* = 683)	0.0	8.9	24.0	13.0	1.3	36.2	0.9	0.4	15.2
Other psychotropic substances	Outpatient (*n* = 79)	0.0	20.3	16.5	24.1	0.0	35.4	0.0	0.0	3.8
	Inpatient (*n* = 1148)	0.3	12.5	23.4	14.8	3.1	30.1	0.4	1.4	13.9
Total	Outpatient (*n* = 5768)	1.5	6.3	45.7	19.0	1.2	21.0	1.4	0.6	3.4
	Inpatient (*n* = 19,037)	1.1	3.9	41.0	20.3	2.2	25.4	1.4	0.6	4.3

F0—organic, including symptomatic, mental disorders; F2—schizophrenia, schizotypal and delusional disorders, F3—mood [affective] disorders; F4—neurotic, stress-related and somatoform disorders; F5—behavioural syndromes associated with physiological disturbances and physical factors; F6—disorders of adult personality and behaviour; F7—mental retardation; F8—disorders of psychological development; F9—behavioural and emotional disorders with onset usually occurring in childhood and adolescence; percentages may not add up to 100% as there are more categories available

### Patients with Co-occurring Mood and Anxiety Disorders

#### Sociodemographic Data

##### Gender

While in the total sample women accounted for just one quarter of all patients (outpatient, 25.5%; inpatient, 26.8%), we found a much higher percentage of women among patients with comorbid mood (outpatient, 41.3%; inpatient, 40.0%) and anxiety disorders (outpatient, 46.0%; inpatient, 47.7%).

##### Age

Patients with comorbid mood (outpatient, 43.8 years; inpatient, 44.7 years) and anxiety disorders (outpatient, 40.7 years; inpatient, 42.4 years) showed an above average age compared to the total sample (outpatient, 38.1 years; inpatient, 41.5 years).

##### Partnership

Independently of having a comorbid diagnosis, about half of all patients were single (outpatient: F3x, 48.4%; F4x, 51.8%; total 50.4%; inpatient: F3x, 53.3%; F4x, 52.8%; total, 53.8%), another 40–45% lived in relationships.

##### Employment Status Before Treatment

Less than half of all patients with comorbid mood disorders were employed before entering treatment (outpatient, 45.5%; inpatient, 40.5%) which is comparable to the total sample (outpatient, 41.1%; inpatient, 36.8%). Patients with comorbid anxiety disorders had the lowest employment rate before treatment (outpatient, 37.5%; inpatient, 35.1%).

#### Distribution of Main Substance Diagnoses

In both settings, more than two thirds of all patients with a comorbid mood (outpatient, 70.7%; inpatient, 81.7%) and anxiety disorder (outpatient, 64.4%; inpatient, 78.4%) were in treatment due to an alcohol-related disorder. Cannabis-related disorders accounted for the second highest proportion (see Table 2). While in inpatient setting the distribution of main substance diagnoses was comparable between patients with comorbid mood or anxiety disorders and the total sample, we found a markedly divergent distribution of main diagnoses among outpatients with a comorbid mood or anxiety disorder and the total outpatient sample. Even though alcohol-related disorders accounted for half of all diagnoses (52.1%), the percentage of opioid, cannabis, stimulants and cocaine diagnoses was much higher in the total outpatient sample. In contrast, main diagnoses due to sedatives were comparatively scarce in the total sample (see Table 2).

**Table 2:** Distribution of main substance diagnosis among patients with mood (F3x) and anxiety (F4x) disorders and the total sample, *percentage* (*N*)

		F3x	F4x	Total
Alcohol	Outpatient	70.7 (2077)	64.4 (809)	52.1 (87,689)
	Inpatient	81.7 (5791)	78.4 (2765)	71.8 (29,724)
Opioids	Outpatient	6.3 (184)	7.1 (89)	14.9 (25,043)
	Inpatient	3.3 (237)	3.7 (130)	6.8 (2805)
Cannabis	Outpatient	7.5 (220)	8.0 (101)	15.4 (25,859)
	Inpatient	4.0 (282)	5.1 (180)	7.1 (2930)
Sedatives	Outpatient	1.9 (57)	3.6 (45)	0.8 (1425)
	Inpatient	1.6 (112)	3.1 (110)	0.9 (375)
Cocaine	Outpatient	1.8 (53)	1.8 (23)	2.3 (3799)
	Inpatient	0.8 (56)	0.5 (18)	1.8 (744)
Stimulants	Outpatient	2.3 (69)	3.4 (43)	5.6 (9479)
	Inpatient	2.0 (145)	2.4 (85)	4.6 (1897)
Other psychotropic substances	Outpatient	0.4 (13)	1.6 (20)	0.6 (1050)
	Inpatient	3.2 (230)	4.0 (142)	3.8 (1587)
Total (*N*)	Outpatient	2937	1256	168,212
	Inpatient	7088	3526	41,395

Percentages may not add up to 100% as there are more categories available

#### Course of Illness and Treatment

##### Duration of the Substance Use Disorder

As demonstrated in Table 3, in outpatients with a comorbid mood disorder, the substance use disorder existed on average for 13.7 years, in patients with a comorbid anxiety disorder for 13.1 years, and therefore up to 2 years longer than in the total sample (11.9 years). In inpatient setting, the duration of the substance use disorder among patients with comorbid mood (13.3 years) and anxiety disorders (13.0 years) was comparable to the total sample (12.4 years).

**Table 3:** Average duration of SUD among patients with mood (F3x) and anxiety (F4x) disorders and the total sample (*in years*)

		Duration of SUD		
		F3x	F4x	Total
Alcohol	Outpatient	16.8	15.6	15.7
	Inpatient	16.9	15.9	16.9
Opioids	Outpatient	11.2	10.6	11.3
	Inpatient	9.7	9.5	9.6
Cannabis	Outpatient	16.5	14.1	11.7
	Inpatient	16.9	15.6	15.0
Sedatives	Outpatient	12.0	11.5	12.5
	Inpatient	10.8	12.3	10.9
Cocaine	Outpatient	13.5	11.7	12.2
	Inpatient	13.3	12.7	11.7
Stimulants	Outpatient	11.1	10.8	9.6
	Inpatient	12.5	12.2	11.2
Other psychotropic substances	Outpatient	14.3	17.5	11.2
	Inpatient	14.4	14.2	12.6
Total	Outpatient	13.7	13.1	11.9
	Inpatient	13.3	13.0	12.4

##### Previous Treatment

In outpatient setting, about two thirds of comorbid patients (mood disorders, 77.7%; anxiety disorders, 76.5%) received at least one previous outpatient or inpatient addiction treatment before entering the current treatment episode, which is a higher proportion than in the total sample (62.8%). Additionally, they showed a markedly higher extent of psychiatric (F3x, 30.6%; F4x, 27.9%; total, 6.5%) and psychotherapeutic pre-treatment (F3x, 18.9%; F4x, 18.0%; total, 4.2%). In inpatient setting, the overall amount of re-treatment among comorbid patients was comparable to the total sample (F3x, 91.0%; F4x, 90.2%; total, 89.0%). The proportion of psychiatric (F3x, 23.7%; F4x, 24.8%; total, 14.2%) and psychotherapeutic pre-treatment (F3x, 17.4%; F4x, 19.9%; total, 10.6%) was also higher among patients with comorbid mood or anxiety disorders.

#### Treatment Characteristics

##### Length of Treatment

The average length of treatment was 269 days for outpatients with comorbid mood disorders and 252 days for outpatients with anxiety disorders (total outpatient sample, 237 days). In inpatient setting, the length of treatment for patients with mood or anxiety disorders (98.5 days) was comparable to the regular length of treatment (93.1 days).

##### Internal Treatment (Within the Facility)

In outpatient setting, internal treatment of patients with comorbid mood or anxiety disorders was compared to the total sample, with counselling and outpatient rehabilitation accounting for the major part (see Table 4). Almost no internal psychiatric (F3x, 2.6%; F4x, 1.9%; total, 0.3%) or psychotherapeutic treatment (F3x, 4.2%; F4x, 5.7%; total, 0.9%) was provided in outpatient treatment. In inpatient setting, almost all patients received internal rehabilitation treatment and a much higher proportion received internal psychiatric (F3x, 9.3%; F4x, 11.1%; total, 9.2%) and psychotherapeutic treatment (F3x, 16.5%; F4x, 18.9%; total, 18.7%) (see Table 4).

**Table 4:** Internal and external treatment of patients with mood (F3x) and anxiety (F4x) disorders and the total sample, *percentage*

Treatment	Outpatient internal			Outpatient external			Inpatient internal			Inpatient external		
	F3x	F4x	Total	F3x	F4x	Total	F3x	F4x	Total	F3x	F4x	Total
Other medical treatment	1.3	0.8	0.5	11.8	12.9	3.7	13.2	16.6	16.2	22.1	27.5	22.4
Detoxification	2.3	0.9	0.8	21.3	21.2	12.1	2.1	1.4	2.4	6.7	6.1	5.0
Outpatient counselling	74.3	77.4	81.6	4.6	4.2	3.1	0.5	0.6	0.9	6.6	6.5	5.9
Outpatient rehabilitation	27.1	23.7	9.7	0.7	0.9	0.4	0.3	0.3	2.0	1.0	0.9	0.8
Inpatient rehabilitation	0.4	0.3	0.3	12.4	14.0	6.1	89.2	88.5	82.5	2.4	2.5	1.7
Outpatient socio-therapy	4.3	3.7	2.0	2.6	3.0	0.8	0.1	0.2	0.1	0.8	1.0	0.6
Psychiatric treatment	2.6	1.9	0.3	23.8	23.9	3.8	9.3	11.1	9.2	4.3	4.2	1.9
Psychotherapeutic treatment	4.2	5.7	0.9	8.4	9.5	1.6	16.5	18.9	18.7	1.6	2.0	1.1
Others	21.2	17.4	16.0	24.0	22.4	14.7	27.8	31.0	33.8	10.8	12.4	10.0

##### External Treatment (in Other Facilities)

In outpatient setting, the extent of complementary external psychiatric and psychotherapeutic treatment (F3x, 32.2%; F4x, 33.4%; total, 5.4%) was higher among patients with comorbid mood or anxiety disorders. In inpatient setting, the amount of external treatment was comparable between patients with comorbid mood or anxiety disorders and the total sample.

#### Treatment Process Outcomes

##### Treatment Termination

In both settings, patients with comorbid mood and anxiety disorders showed a high level of treatment adherence. In outpatient setting, 69.3% of all patients with mood disorders and 69.2% of patients with anxiety disorders completed their treatment regularly (total, 63.5%). Among inpatients with comorbid mood (85.6%) and anxiety disorders (84.2%), the proportion of regular treatment termination was even higher than in outpatient setting (total, 80.4%).

##### Treatment Outcome Assessment

**Fig. 1:**
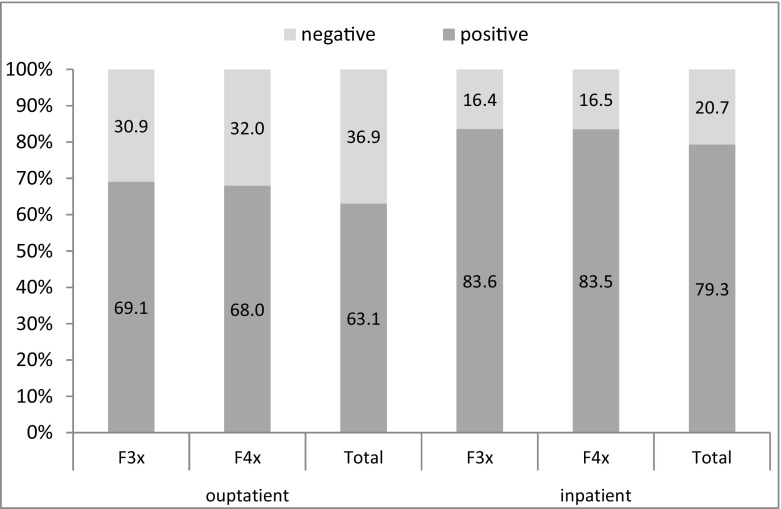
Treatment process outcome for patients with mood (*F3x*) and anxiety (*F4x*) disorders and the total sample, *percentage*

In both, outpatient and inpatient setting, patients with comorbid mood or anxiety disorders showed highly successful treatment process outcomes. In outpatient treatment, 69.1% of all patients with mood and 68.0% of those with anxiety disorders showed an improvement of their substance-related problems after the end of treatment (total, 63.1%). In inpatient setting, actually 83.6% of all patients with comorbid mood and 83.5% with anxiety disorders were assessed as successful after treatment (total, 79.3%) (see Fig. 1).

#### Supply Structures

##### Referral into Treatment

Outpatients with comorbid mood or anxiety disorders showed comparable referral routes into treatment as the total sample (see Table 5). Only referrals by hospitals or rehabilitation centres were slightly more often, whereas referrals due to legal authorities were scarce. Inpatients with comorbid mood or anxiety disorders experienced similar ways into treatment as the total sample. The majority was referred by outpatient counselling centres, hospitals, rehabilitation centres or purchase and service providers.

**Table 5:** Referral into treatment and co-operation with other services among patients with mood (F3x) and anxiety (F4x) disorders and the total sample, *percentage*

	Referral into treatment						Co-operation during treatment					
	Outpatient			Inpatient			Outpatient			Inpatient		
	F3x	F4x	Total	F3x	F4x	Total	F3x	F4x	Total	F3x	F4x	Total
Self/family	39.4	40.5	46.1	4.5	6.1	4.9	25.2	27.6	18.2	43.6	39.5	39.8
Medical/psychotherapeutic care	9.0	8.6	7.5	1.8	1.6	1.2	27.6	29.1	14.4	22.2	26.4	22.9
Addiction counselling	7.9	6.9	3.5	56.7	57.9	58.8	5.7	4.7	3.9	62.9	65.4	56.5
Hospital unit	13.8	11.6	8.4	20.9	17.5	17.7	19.5	20.0	10.6	11.9	12.9	8.3
Adaption	0.3	0.5	0.1	0.1	0.1	0.0	0.3	0.8	0.6	9.0	9.5	9.4
Inpatient rehabilitation	10.8	10.5	4.0	5.6	6.4	6.5	21.9	24.4	11.8	5.3	6.3	6.5
Legal autonomy	1.6	2.1	7.8	0.1	0.1	0.3	3.2	4.3	10.0	4.3	4.7	10.5
Social service/prison	0.4	0.2	1.8	0.2	0.3	1.3	–	–	–	–	–	–
Employment agency	1.0	2.0	3.0	0.1	0.2	0.2	3.8	8.4	5.8	17.2	15.4	20.4
Purchaser/service provider	2.8	2.1	1.4	3.4	2.7	2.9	37.5	39.4	19.9	45.5	42.3	40.9
Others	17.2	18.6	18.0	6.7	9.1	7.7	52.0	66.5	34.6	85.3	88.8	81.3

##### Co-operations with Other Services

In outpatient setting, more co-operations with other services, like medical or psychotherapeutic care, hospitals, rehabilitation centres or service providers, existed in the treatment of patients with comorbid mood or anxiety disorders (see Table 5). In inpatient setting, co-operations with other services were generally more frequent than in outpatient setting, even though the level of co-operations was comparable in the treatment of comorbid inpatients.

##### Referral After Treatment

After treatment, outpatients with comorbid mood (50.9%) and anxiety disorders (49.3%) were more often referred to other health care services than the total sample (32.8%). A substantially higher number was referred to medical or psychotherapeutic care (F3x, 24.7%; F4x, 24.2%; total, 9.2%). Although in inpatient setting the extent of referrals was relatively high in general (F3x, 79.1%; F4x, 79.0%; total, 76.8%), patients with comorbid mood or anxiety disorders were referred even more frequently to medical or psychotherapeutic services after treatment (F3x, 27.0%; F4x, 28.1%; total, 20.0%).

## Discussion

In the following, results will be discussed with regard to their implications for the supply situation of patients with co-occurring mental disorders in substance abuse treatment.

### Prevalence of Comorbid Psychiatric Diagnosis

The distribution of additional psychiatric diagnoses, with mood, anxiety and personality disorders as the most prevalent comorbid disorders, is mainly consistent with the results of earlier studies (Adamson et al. 2006; Flensborg-Madsen et al. 2009; Langas et al. 2011). Also, the high co-occurrence of mood and anxiety disorders in patients with alcohol or sedative use (Burns and Teesson 2002; Do and Mezuk 2013; Goodwin et al. 2004; Merikangas et al. 1998; Smith and Book 2010) as well as the high association between problems due to illegal substances with comorbid schizophrenia or personality disorders and disorders with onset in childhood and adolescence confirms previous results (Biedermann et al. 1995; Gouzoulis-Mayfrank 2007; Lieb et al. 2010; Salo et al. 2011). Whereas in inpatient treatment, the overall comorbidity rate corresponds mainly to the findings of the literature (Adamson et al. 2006; Flensborg-Madsen et al. 2009), the low frequency of additional diagnoses in outpatient setting indicates an underdiagnosing of comorbid mental disorders. This may be due to structural differences, like limited personal resources (mainly social workers) in outpatient setting and different assignments that, in outpatient setting, do normally not require a standardised diagnostic assessment and even less the treatment of additional mental disorders.

### Patients with Comorbid Mood or Anxiety Disorders

The high proportion of alcohol use disorders and the lower percentage of diagnoses due to illegal substances in the sample of patients with comorbid mood and anxiety disorders are consistent with previous results. With regard to sociodemographic characteristics, the higher proportion of women in this sample may be explained in terms of a generally higher probability of women having a comorbid mental disorder (Brady et al. 1993; Zilberman et al. 2003) and a mood or anxiety disorder in general (Jacobi et al. 2014; Kessler et al. 1994). Altogether patients with comorbid mood or anxiety disorders do not show higher levels of psychosocial strains, like higher unemployment or general poorer social circumstances, which could have been expected as a result of the additional burden due to the comorbid disorder. Maybe this effect is moderated by the high extent of “legal” main diagnoses in these patients, which are generally associated with better social circumstances (Brand et al. 2015).

The longer duration of the substance use disorder and the higher proportion of retreated patients with comorbid mood and anxiety disorders are consistent with earlier studies and demonstrate that individuals with comorbidity show a more complicated course of illness and treatment (Kessler et al. 1996).

The higher extent of complementary external psychiatric and psychotherapeutic treatment and of co-operations with psychiatric or medical practices in the treatment of patients with a known comorbid mood or anxiety disorder indicates that especially in outpatient setting the treatment of comorbid patients is frequently supplemented by other health care services to enable an optimal treatment. In inpatient setting, the slighter differences in the treatment of comorbid patients and the lesser extent of co-operations with other services may not be interpreted as a lack of additional treatment. Here, the overall higher extent of internal treatment argues for the fact that complementary psychiatric treatment is mainly an integrative and regular component of treatment that is available for all patients in the same way. As mentioned above, the divergent findings in inpatient and outpatient treatment of comorbid patients may likely be due to structural differences. Thus, it can be assumed that it is more unlikely for outpatient facilities to provide additional treatment (besides addiction care) within the treatment centre, whereas in inpatient setting more resources are available to provide complementary treatment within the facility. Nevertheless, findings indicate that patients with comorbid psychiatric diagnoses receive additional treatment in both settings, which is further reflected by the high treatment adherence and positive treatment outcomes among patients with comorbid diagnoses.

### Limitations

In general, the structural differences between treatment centres, included in this study, make it difficult to receive consistent findings and to draw universal conclusions. To address this concern, results were reported separately for inpatient and outpatient settings. The major limitation of this study lies in the fact that no statement can be made on the treatment of patients with comorbid mental disorders that are not yet identified (i.e. not having a diagnosis). Therefore, it remains unclear if the existence of a diagnosis alone leads to the provision of additional treatment or if psychiatric problems are also incorporated in treatment if no corresponding diagnosis exists. To figure this out, a specific comparison between comorbid patients with and without diagnosis would be inevitable, although it may turn out difficult to identify relevant patients. Another limitation involves the treatment process outcome, which is only assessed with regard to the problematic substance use. Although this is adequate within the context of Substance Abuse Treatment, it allows no statement on the status of the mental disorder. Another weakness lies in the lack of information on the time-frame of the comorbid psychiatric diagnosis (lifetime, last year or last month), which is not assessed in the standard documentation form and might differ with regard to diagnoses. In spite of the supposed underestimation of comorbid diagnosis in outpatient setting, the large sample size of 3703 patients with a documented comorbid F3x or F4x diagnosis can be considered as sufficient to describe this sample and to draw valid conclusions.

## Conclusion

Findings of this investigation provide insight into the actual management of comorbid mental disorders in German addiction care and delineate strengths and weaknesses in the treatment of patients with comorbidity.

For conclusion and with regard to our initial study aims, findings demonstrate that in Germany in general necessary structures and resources are available to provide appropriate treatment for patients with comorbidity. Patients with a known comorbid psychiatric diagnosis receive complementary treatment for their additional problems in inpatient as well as in outpatient treatment. Furthermore, they highly benefit from this treatment, which is reflected in good treatment adherence and positive treatment process outcomes.

This indicates that the knowledge of an existing additional mental disorder has influence on treatment planning and the health care provision of affected patients. Hence, findings of this study highlight the importance of a sufficient consideration and diagnostic assessment of comorbid mental disorders in substance abuse treatment. This is especially important for outpatient setting where capabilities are generally more restricted and a diagnostic assessment and treatment of comorbid mental problems is usually not intended. Therefore, the application of available short, efficient and validated screening tools for comorbid disorders (Mestre-Pintó et al. 2014; Hasin et al. 1996) should be further promoted. But keeping in mind the limited resources in addiction care, interventions like trainings for staff members (Hunter et al. 2005; Marshall and Deane 2004; Minkoff and Cline 2004) that focus on a better detection and management of comorbid mental disorders seems worthwhile and may contribute to enhance the sensitivity for comorbid mental disorders. Furthermore, to encounter the common barriers, co-operations and networks with mental health care should be focused and further expanded to assure an adequate and effective treatment for patients with comorbidity.
